# Forecasting global monthly cotton prices: the superiority of NNAR models over traditional models

**DOI:** 10.3389/frai.2025.1628744

**Published:** 2025-12-04

**Authors:** Rumita Limbu Sanwa, Raksha Khadka, Yeon Nain Chi

**Affiliations:** Department of Agriculture, Food, and Resource Sciences, University of Maryland Eastern Shore, Princess Anne, MD, United States

**Keywords:** NNAR, hybrid models, cotton price forecasting, time series, predictive model

## Abstract

Accurate forecasting of agricultural commodity prices is essential for informed decision-making by farmers, traders, and policymakers. This study evaluates and compares the predictive performance of traditional statistical and machine learning models in forecasting global monthly cotton prices. Price volatility and nonlinear patterns in cotton markets present challenges for conventional models such as the Auto Regressive Integrated Moving Average (ARIMA), which often fail to capture complex dynamics. The novelty of this research lies in systematically comparing traditional statistical models (ARIMA, ETS, STL, TBATS, Theta), machine learning models (Neural Network Auto-Regressive [NNAR]), and hybrid approaches to determine the best forecasting tool. Performance was evaluated using Root Mean Square Error (RMSE), Mean Error (ME), Mean Absolute Error (MAE), Mean Percentage Error (MPE), and Mean Absolute Percentage Error (MAPE). Results revealed that the NNAR (26, 1, 14) [12] model outperformed all models, achieving the lowest RMSE (1.16383774), MAE (0.832275572), and MAPE (1.19%), indicating high predictive accuracy and minimal bias. The 30-month forecast for cotton prices using the NNAR model indicates fluctuations between approximately $0.66 and $0.74 per pound, following a cyclical pattern without a clear long-term trend. These findings highlight the strength of advanced machine learning techniques, particularly NNAR, in capturing complex nonlinear patterns, improving forecasting reliability, and supporting effective decision-making in volatile cotton markets. This study provides practical insights for stakeholders seeking to anticipate cotton price changes and make informed decisions in the global market.

## 1 Introduction

Cotton *(Gossypium* spp.*)* is an important cash crop cultivated commercially in more than 50 countries and plays a vital role in supporting livelihoods worldwide (Khadi et al., 2010). Often referred to as “white gold,” cotton contributes substantially to foreign exchange earnings in many countries (Khan et al., 2020). Its importance extends far beyond the farm; cotton is at the heart of global industries, economies, and cultures (Jabran et al., 2019). However, its price is highly variable, influenced by seasonal changes, regional differences, global market conditions, and broader economic trends. For farmers and stakeholders in the textile supply chain, such variability creates substantial uncertainty in decision-making (Darekar and Reddy, 2017).

Price volatility in cotton markets poses a serious challenge for producers, traders, and policymakers. Inaccurate price expectations can result in inefficient resource allocation, poor risk management, and financial instability among farmers (Hudson, 1998; Wang, 2023). As a key raw material for the textile industry, cotton faces production and price uncertainties, posing challenges for farmers. Since prices are shaped by local and international supply and demand, accurate price forecasting has become increasingly important, especially following market liberalization, which has led to more frequent price fluctuations and reduced predictability in cotton farming. In this context, timely and accurate price forecasts play an important role in enhancing market efficiency and supporting the financial stability of cotton producers (Darekar and Reddy, 2017; Kumar et al., 2024).

Accurate price forecasts are crucial for strategic planning in agriculture. It works like a dynamic filter, using past price data to predict future prices. Accurate forecasts help farmers and industries plan their farming activities and budgets, which often depend on expected future prices (Chi, 2021). They enable farmers to optimize crop choices and marketing strategies, while industries and policymakers use them to ensure supply chain stability and maintain competitiveness in global markets (Sun et al., 2023). Price shifts can significantly impact not just livelihoods and food affordability, but also the broader economy and supply chains that rely on affordable raw materials (Wang, 2023). It affects not only what people pay for food, but also how farmers plan their production. Given cotton’s pivotal role in the global textile industry, advancements in forecasting models contribute not only to agricultural development but also to economic stability and food security (Zhang et al., 2020). As Hudson (1998) noted, when producers have access to accurate price information, they are better able to decide what and when to produce or sell. This knowledge benefits not only the farmers, but also plant breeders, ginners, merchants, and textile mills who depend on predictable prices to manage supply chains and production costs.

## 2 Literature review

### 2.1 Traditional time series models for cotton price forecasting

Cotton prices are inherently volatile, making reliable forecasting models essential for supporting informed decision-making. The Auto Regressive Integrated Moving Average (ARIMA) model, originally proposed by Box and Jenkins (1970), has emerged as a widely used and practical statistical approach for modeling and predicting time series data that follows regular and continuous patterns. Its ability to capture trends, seasonality, and autocorrelations in historical price data makes it particularly suitable for forecasting in agricultural markets such as cotton, where structured, periodic fluctuations are common (Weng et al., 2019). However, its performance tends to decline when dealing with more complex or nonlinear price movements, as noted by Liu (2025). Similarly, Wang et al. (2013) emphasized ARIMA’s strengths as a linear forecasting tool for economic time series but also acknowledged its limitations in capturing sudden market fluctuations. To address these limitations, Other linear forecasting models have also been applied to capture trends and seasonal patterns, including the Exponential Smoothing State Space (ETS) model (introduced by Hyndman et al., 2008), the Seasonal and Trend decomposition using Loess (STL) model (introduced by Cleveland et al., 1990), and the Theta model (introduced by Assimakopoulos and Nikolopoulos, 2000). For time series with multiple or irregular seasonal patterns, the *TBATS* model (*Trigonometric, Box-Cox Transformation, ARMA Errors, Trend, and Seasonal components*) is particularly effective (De Livera, 2010; Hyndman and Athanasopoulos, 2021). It is designed to handle time series with multiple seasonal patterns, irregular cycles, or high-frequency seasonality. De Livera (2010) showed that TBATS can outperform more traditional models when dealing with such complex seasonal behavior. Later, Hyndman and Athanasopoulos (2021) expanded on its capabilities, showing how TBATS can be applied to a wide range of forecasting problems with irregular or multi-seasonal data patterns.

### 2.2 Machine learning approaches

To overcome these limitations of traditional linear models, researchers have increasingly turned to machine learning (ML) methods such as artificial neural networks (ANNs), which can model complex, nonlinear relationships in economic data (Patel et al., 2020; Raghav et al., 2022). Neural networks have proven to be powerful tools for time series forecasting due to their ability to model complex, nonlinear relationships. Zhang and Qi (2005) demonstrated that they outperform traditional statistical models, particularly when handling time series data with irregular fluctuations and unpredictable patterns. Reza and Debnath (2025) further demonstrated that the *Neural Network Autoregressive (NNAR)* model, first introduced by Zhang (2003), outperformed the ARIMA model in forecasting accuracy. Their study showed that NNAR achieved higher predictive accuracy, as measured by R-squared, and produced lower forecasting errors, highlighting its ability to capture complex and nonlinear patterns in time series data. Similar results were reported by Melina et al. (2024), who found that the NNAR model consistently outperformed ARIMA in price forecasting tasks. The strength of the NNAR model lies in its flexibility. Unlike traditional linear models, it does not assume linearity or normality, making it particularly effective for capturing the nonlinear dynamics often present in agricultural commodity prices. This adaptability makes NNAR valuable for improving forecast reliability in complex and rapidly changing market environments. Recent research has focused on combining neural network models with traditional forecasting methods to enhance accuracy, particularly for datasets exhibiting linear and nonlinear characteristics. Almarashi et al. (2024a) demonstrated that the NNAR model outperformed other models, achieving the best evaluation metrics in time series forecasting. Among various artificial neural network (ANN) architectures, the single hidden layer ANN is frequently used for time series forecasting due to its simplicity and effectiveness (Zhang et al., 1998). A real-world time series may exhibit both linear and nonlinear patterns.

### 2.3 Hybrid forecasting models

Hybrid forecasting models that combine statistical and machine learning approaches have been shown to enhance predictive performance by combining the strengths of both linear and nonlinear methods. Zhang (2003) introduced the ARIMA-ANN hybrid model, demonstrating that neural networks can effectively complement ARIMA by capturing nonlinear structures that traditional models fail to represent. This study laid the groundwork for the development of neural network autoregressive (NNAR) models, which apply feed-forward neural networks for time series forecasting. Subsequent studies, such as Wang et al. (2013), Kumar and Thenmozhi (2014), and Babu and Reddy (2014), provided further evidence that hybrid approaches outperform standalone models across financial and economic datasets. More recently, Sun et al. (2023) reaffirmed the potential of hybrid and neural-based approaches in improving the accuracy and robustness of cotton price forecasting. Collectively, these studies support the use of NNAR models as a robust extension of ARIMA-ANN frameworks for modeling complex time series dynamics.

Another hybrid methodology combining linear and nonlinear exponential smoothing models from innovation state space (ETS) with artificial neural networks (ANN) was proposed (Panigrahi and Behera, 2017). The model demonstrated superior forecast accuracy due to their ability to capture diverse linear and nonlinear patterns, leveraging the strengths of both ETS and ANN in modeling. In another approach, Jaiswal et al. (2022) introduced a hybrid model that combines Seasonal-Trend Decomposition using Loess (STL) with an Extreme Learning Machine (ELM), called STL-ELM. This method is particularly useful for forecasting agricultural prices, which often show seasonal and nonlinear trends. Their study found that STL-ELM captured these complex patterns more accurately than traditional methods, improving forecasting performance for challenging real-world data.

Advances in statistical modeling highlight the need for robust and reliable methods in prediction and estimation. For example, Ahmad et al. (2025a) developed an auxiliary variables-based estimator to improve population distribution estimates under stratified sampling and non-response, and Ahmad et al. (2025b) proposed an unbiased ratio estimator for distribution functions in complex sampling settings. While these studies are not directly focused on agricultural markets, they underline the importance of methodological innovation in improving predictive accuracy. Inspired by this principle, the present study evaluates linear, nonlinear, and hybrid forecasting approaches to improve the reliability of global cotton price predictions.

This study seeks to address the following key research questions:

1. How accurately do traditional models such as ARIMA, ETS, Theta, and TBATS perform in forecasting global monthly cotton prices?
2. To what extent can machine learning models such as Neural Network Auto-Regressive (NNAR) improve forecasting performance compared to traditional statistical models?
3. Do hybrid models that combine statistical and machine learning techniques provide more reliable forecasts by capturing both linear and nonlinear patterns in cotton price forecasting?

The primary objectives of this research are:

- To identify and compare the most suitable time series models in forecasting monthly global cotton prices.
- To assess the effectiveness of hybrid forecasting approaches in enhancing prediction reliability for complex agricultural time series.
- To provide practical insights for cotton industry stakeholders by improving the reliability of price forecasts.

### 2.4 Summary and research gap

Accurate forecasting of cotton prices is essential for producers, traders, and policy makers, yet the underlying time series often exhibit a mixture of linear trends, seasonality, and nonlinear fluctuations. Forecasting methods have evolved from traditional statistical models to advanced machine-learning approaches, each with distinct strengths and limitations. Linear models such as ARIMA and ETS capture autoregressive and seasonal components effectively but tend to perform poorly on irregular or nonlinear patterns (Wang et al., 2013; Weng et al., 2019). Conversely, neural-network-based methods, particularly the Neural Network Auto-Regressive (NNAR) model, are well-suited to modelling nonlinear dynamics and have outperformed ARIMA in various applications (Reza and Debnath, 2025; Almarashi et al., 2024b). However, empirical and methodological studies indicate that NNAR and related models may underperform when a time series contains a dominant linear structure, motivating hybrid strategies that model linear and nonlinear components separately (Zhang, 2003; Khandelwal et al., 2015).

Hybrid frameworks such as ARIMA-ANN, ETS-ANN, and STL-ELM have demonstrated improved forecast accuracy in diverse fields, including economics, stock returns, and agricultural commodities (Wang et al., 2013; Kumar and Thenmozhi, 2014; Sun et al., 2023; Panigrahi and Behera, 2017; Jaiswal et al., 2022). Yet, despite these advances, few studies have systematically compared linear, nonlinear, and hybrid approaches within a single framework for cotton price forecasting. Existing comparative work (e.g., Kumar and Thenmozhi, 2014; Babu and Reddy, 2014) has focused mainly on financial datasets, limited regions, or small samples and generally omitted NNAR alongside hybrid models.

Accordingly, this study addresses this gap by systematically evaluating and comparing the forecasting performance of ARIMA, ETS, Theta, TBATS, STL, NNAR, and hybrid approaches to identify the most effective models for predicting global cotton prices.

## 3 Materials and methods

### 3.1 Data

The data used in this study, the monthly global cotton price (PCOTTINDUSDM) from January 1990 to January 2025 (Units: U. S. Cents per Pound, Not Seasonally Adjusted), is sourced from the International Monetary Fund via FRED, the Federal Reserve Bank of St. Louis.^1^ The *forecast Hybrid* package in R (version 4.4.2) was used to implement a hybrid model that integrates six forecasting methods: auto.arima, ets, thetam, nnetar, stlm, and tbats. Equal weights (i.e., each model contributed equally to the final forecast) and weights based on cross-validation errors for higher accuracy were assigned to each model. The set.seed () function ensured reproducibility of results, and forecasts for the next 30 months were generated using the forecast () function. Model performance was assessed with standard error metrics, including Mean Absolute Error (MAE), Root Mean Squared Error (RMSE), Mean Percentage Error (MPE), Mean Absolute Percentage Error (MAPE), and Mean Error (ME). Forecasts were visualized using the plot () function to compare the predicted values across different models and hybrids. This graphical representation highlighted the relative performance of the models in capturing trends and seasonal patterns.

### 3.2 Methodology

We employed multiple forecasting models to model the monthly price of cotton, including a hybrid forecasting approach that combines linear and nonlinear time series models. The following models were utilized for prediction:

#### 3.2.1 Autoregressive integrated moving average (ARIMA)

The ARIMA model is widely used for forecasting, incorporating past values (autoregression), differencing to achieve stationarity (d), and past forecast errors (moving average) (Box and Jenkins, 1970). The (d-differencing order) represents the number of differencing steps required to transform a time series into a stationary one. To identify whether the series is stationary and determine the appropriate d value, we applied the Augmented Dickey-Fuller (ADF) test proposed by Dickey and Fuller (1981). However, selecting the optimal parameters (p-autoregressive order), (d-differencing order), and (q-moving average order) can be complex and require statistical expertise. In practice, automatic forecasting for large univariate time series is often needed, especially in business contexts. According to Hyndman and Athanasopoulos (2018), the auto. Arima () function in R utilizes the Hyndman-Khandakar algorithm (Hyndman and Khandakar, 2008). This algorithm simplifies model selection by integrating unit root tests to determine differencing requirements, minimizing the corrected Akaike Information Criterion (AICc) for model selection, and applying Maximum Likelihood Estimation (MLE) to optimize parameters, ensuring the most suitable ARIMA model is identified.

The general formula for the ARIMA (p, d, q) model, based on the foundational work of Box and Jenkins (1970), is expressed as:


$$\phi_{p}(B){\left(1-B\right)}^{d}y_{t}=C+\theta_{q}(B)\epsilon_{t}$$ (1)

Where:

-
B is the backshift operator $B\;y_{t}=y_{\left\{t-1\right\}}$
-
$\phi_{p}(B)\;$ represents the autoregressive (AR) polynomial of order p:

$$\phi_{p(B)}=1-\phi_{1}B-\phi_{2}B^{2}-\ldots -\mathrm{\phi p}\;B^{p}$$

-
${\left(1-B\right)}^{d}$ represents the differencing in order d.
- C is a constant term
-
θq(B) represents the moving average (MA) polynomial of order q:

$$\theta_{q(B)}=1-\theta_{1}B-\theta_{2}B^{2}-\ldots -\theta_{q}B^{q}$$

-
$\epsilon_{t}$ is the error term, which is assumed to be white noise.

#### 3.2.2 Seasonal autoregressive integrated moving average model (SARIMA) model

Considering the seasonal pattern exhibited by the monthly cotton price, a seasonal process may be considered; therefore, the ARIMA model will become a Seasonal Autoregressive Integrated Moving Average (SARIMA) model. The SARIMA model extends the ARIMA model by incorporating seasonal effects, making it well-suited for analyzing such time series data. Khadka and Chi (2024) demonstrated that the SARIMA model effectively captures seasonal patterns and trends in agricultural price data.

The SARIMA model is denoted as ARIMA (p, d, q) (P, D, Q) S and has the following specification based on the backshift operator

- *(p, d, q)* denote the non-seasonal autoregressive order, differencing order, and moving average order.
- *(P, D, Q)* represent the seasonal components of autoregression, differencing, and moving average.
- *s* refers to the length of the seasonal cycle (e.g., *s = 12* for monthly data).

This formulation enhances the model’s flexibility in capturing complex patterns by incorporating seasonal and non-seasonal behaviors within the same structure (Box et al., 2015). The general form of the SARIMA model using the backshift operator B is Chang et al. (2012):


$$\Phi_{P}(B^{s})\phi_{p}(B){\left(1-B\right)}^{d}{\left(1-B^{s}\right)}^{D}\;y_{t}=C+\Theta_{Q}(B^{s})\theta_{q}(B)\epsilon_{t}$$ (2)

Where,

$\phi_{p}(B)$ : non-seasonal autoregressive polynomial.

$\theta_{q}(B)$ : non-seasonal moving average polynomial.

$\Phi_{P}(B^{s})$ : seasonal autoregressive polynomial.

$\Theta_{Q}(B^{s})$ : seasonal moving average polynomial.

${\left(1-B\right)}^{d}$ : non-seasonal differencing.

${\left(1-B^{s}\right)}^{D}:$ seasonal differencing.

C : constant (intercept term).

$\epsilon_{t}\;$ : white noise error term

#### 3.2.3 Neural network autoregression (NNAR) model

Artificial neural networks are forecasting techniques inspired by simplified mathematical representations of the brain’s functioning, enabling them to capture complex nonlinear relationships between variables. The NNAR (Neural Network Autoregressive) model integrates traditional autoregressive (AR) models with artificial neural networks (ANN) to capture nonlinear relationships in time series data. This model utilizes lagged values of the series as input variables for a feed-forward network, where a hidden layer introduces nonlinearity, and the output layer generates predictions for future observations. The NNAR framework is designed to detect intricate patterns in data, making it highly suitable for forecasting scenarios involving nonlinear relationships (Hyndman and Athanasopoulos, 2021).

The NNAR (p, k) model, which uses lagged values as inputs and k hidden neurons, can be represented as:


$$Y_{t}=f(\sum_{\left\{i=1\right\}}^{\left\{p\right\}}w_{i}Y_{\left\{t-i\right\}}+b)$$ (3)

Where:

-
$Y_{t}$ is the predicted value at time t,
-
f(·) is the non-linear activation function for the hidden layer
- The sum represents the weighted combination of the lagged inputs
-
$w_{i}$ are the weights associated with the lagged values $Y_{\left\{t-i\right\}}$
-
b is the bias term for the hidden layer.

The NNAR (p,P,k )m model is represented with p as the number of input lags, P as the seasonal lags, k as the number of neurons in the hidden layer, and m as the seasonal period length (Hyndman and Athanasopoulos, 2018). The mathematical representation of the NNAR (p, P, k) m model is given as follows:


$$\begin{array}{l} y_{t}=f(y_{\left\{t-1\right\}},y_{\left\{t-2\right\}},\ldots ,y_{\left\{t-p\right\}},y_{\left\{t-m\right\}},\\ y_{\left\{t-2m\right\}},\ldots ,y_{\left\{t-\mathrm{Pm}\right\}},\theta )+\epsilon_{t} \end{array}$$ (4)

Here, f represents the neural network with k hidden nodes in a single layer, and ε_t_ is the residual series.

The NNAR (p, P, k) m model is implemented in R through the nnetar () function in the “forecast” package, which automatically selects appropriate values for p, P, and k based on criteria like the Akaike Information Criterion (AIC). For seasonal time series, it sets *p* = 1 and selects p based on the optimal linear model fitted to the seasonally adjusted data. The default number of hidden nodes k is calculated as k = (p + P + 1)/2, rounded to the nearest integer (Hyndman and Athanasopoulos, 2021).

#### 3.2.4 Exponential smoothing state space model (ETS)

Exponential smoothing methods generate forecasts by applying exponentially decreasing weights to past observations, prioritizing more recent data (Holt, 1957; Brown, 1959; Winters, 1960). This methodology enables efficient and reliable predictions across diverse time series, making it particularly advantageous for industrial applications. As described by Hyndman et al. (2008), ETS models systematically account for various patterns in time series data while allowing for different combinations of error structures, trend components, and seasonal adjustments. The method selection is typically determined by identifying the main components of the time series, such as trend and seasonality, and how these components are incorporated into the smoothing method (e.g., additive, damped, or multiplicative). Each model includes a measurement equation representing the observed data and state equations describing how the unobserved components (level, trend, seasonal) evolve over time. As a result, these models are referred to as state space models (Hyndman and Athanasopoulos, 2018).

The general ETS framework is given as ETS (A, N, N), ETS (M, Ad, N), etc., where:

- Error (E): Additive (A) or Multiplicative (M)
- Trend (T): None (N), Additive (A), Multiplicative (M), or Additive damped (Ad)
- Seasonality (S): None (N), Additive (A), or Multiplicative (M)

Mathematical Formulation of ETS (M, Ad, N) (Hyndman and Athanasopoulos, 2018):


$$l_{t}=\alpha y_{t}+(1-\alpha )(l_{\left\{t-1\right\}}+\phi \;b_{\left\{t-1\right\}})$$

$$b_{t}=\beta (l_{t}-l_{\left\{t-1\right\}})+(1-\beta )b_{\left\{t-1\right\}}$$

$${\hat{y}}_{t+h}=\ell_{t}+h\;b_{t}$$ (5)

Where:

-
$l_{t}\;$ = level at time t,
-
$b_{t}$ = trend at time t,
-
$y_{t}$ = observed value at time t,
- *α*, *β*, ϕ = smoothing parameters
-
${\hat{y}}_{t+h}$ = forecasted value at time t + h
- h = forecast horizon

No seasonal equation exists since this model has no seasonal component (N). The model ETS (M, Ad, N), used in the given forecast, includes a multiplicative error structure, an additive damped trend, and no seasonality. Pegels (1969) introduced a method that incorporated a multiplicative trend. This approach was later expanded by Gardner (1985) to include methods with an additive-damped trend.

#### 3.2.5 TBATS (trigonometric, Box-Cox, ARMA, trend, seasonal)

The BATS model (Box-Cox, ARMA, Trend, Seasonal) is a time series forecasting method for complex seasonal and trend patterns. It stabilizes variance using Box-Cox transformations, models autocorrelations with ARIMA, captures long-term trends, and incorporates seasonal components for periodic fluctuations. Additionally, it uses trigonometric functions to account for non-annual and multiple seasonalities, making it suitable for non-linear data.

The TBATS model introduced by De Livera (2010) extends Exponential Smoothing State Space Models (ETS) to handle multiple seasonality, including integer, non-integer, and dual calendar effects. It uses a state-space framework based on the innovations approach, enhancing flexibility for complex seasonal structures. TBATS can model multiple seasonal periods and long seasonal cycles, with Fourier terms enabling it to capture non-linear seasonality alongside traditional effects.

The framework offers several benefits: (i) a larger parameter space for improved forecasts (Hyndman et al., 2008), (ii) support for both nested and non-nested seasonal components; (iii) handling of nonlinear features standard in real-time series; (iv) accounting for autocorrelation in residuals; and (v) a more straightforward, more efficient estimation procedure. In addition, it is shown that the TBATS models can be used to decompose complex seasonal time series into trend, seasonal, and irregular components. In decomposing time series, the trigonometric approach has several important advantages over the traditional seasonal formulation.

A TBATS (p, $\omega_{i}$ , $\phi ,m_{i}\;)\;$ consists of:

- T: Trigonometric seasonality (Fourier terms).
- B: Box-Cox transformation.
- A: ARMA errors.
- T: Trend.
- S: Seasonal components.

The model is formulated as follows:

a. Box-Cox transformation

$$y_{t}^{\left\{(\lambda )\right\}}=\{\;\frac{\left(y_{t}^{\lambda }-1\right)}{\lambda \;},\log(y_{t}),\text{if}\;\lambda \neq 0\;\text{and}\;\text{if}\;\lambda =0\}$$

Where λ is the Box-Cox transformation parameter.

b. Local trend component

$$\ell_{t}=\ell_{\left\{t-1\right\}}+\phi b_{\left\{t-1\right\}}+\alpha \varepsilon_{t}$$

$$b_{t}=\phi b_{\left\{t-1\right\}}+\beta \varepsilon_{t}$$

Where:

-
$\ell_{t}\;$ is the level,
-
$b_{t}$ is the trend,
-
ϕ is the damping parameter,
-
α,β smoothing parameters,
-
$\varepsilon_{t}\;$ is the error term.

c. Trigonometric seasonal component

For each seasonal period $m_{i}$ , define:


$$s_{\left\{t,i\right\}}=\sum_{\left\{k=1\right\}}^{\left\{K_{i}\right\}}\gamma_{\left\{i,k\right\}}\cos(\frac{\left\{2\mathrm{\pi k}\;t\right\}}{\left\{m_{i}\right\}})+\omega_{\left\{i,k\right\}}\sin(\frac{\left\{2\mathrm{\pi k}\;t\right\}}{\left\{m_{i}\right\}})$$

Where:

-
$s_{\left\{t,i\right\}}$ : seasonal effect at time t, for seasonality i
-
$\gamma_{\left\{i,k\right\}},\omega_{\left\{i,k\right\}}$ : Parameters
-
$m_{i}$ : seasonal period for component i
-
$K_{i}$ : number of harmonics for seasonality i

d. ARMA error component

$$\varepsilon_{t}=\sum_{\left\{j=1\right\}}^{\left\{p\right\}}\phi_{j}\varepsilon_{\left\{t-j\right\}}+\sum_{\left\{j=1\right\}}^{\left\{q\right\}}\theta_{k}\eta_{\left\{t-k\right\}}+\mathrm{\eta t}$$

Where:

-
$\phi_{j}$ are AR terms,
-
$\theta_{k}$ are MA terms,
-
ηt is white noise.

The full TBATS model combines these components:


$$y_{t}^{\left\{(\lambda )\right\}}=\ell_{t}+s_{\left\{t,1\right\}}+s_{\left\{t,2\right\}}+\ldots +s_{\left\{t,I\right\}}+\varepsilon_{t}$$ (6)

This allows TBATS to handle multiple seasonal periods, long seasonal cycles, and damping trends, making it highly effective for complex and irregular seasonal data.

#### 3.2.6 Theta model

The Theta model, introduced by Assimakopoulos and Nikolopoulos (2000), enhances forecasting accuracy by decomposing a time series into two or more Theta lines, which are independently extrapolated before being recombined to produce forecasts. The model’s core principle is decomposing the series into linear and curvature-adjusted components. It performed well using Theta = 0 (linear trend) and Theta = 2 (enhanced local curves), especially for monthly and microeconomic data.

One common version of the formula used in this model is:


$$y_{t}=\theta_{1}\cdot y_{t}+\theta_{2}\cdot {\left\{\text{Trend}\right\}}_{t}$$ (7)

Where:

-
$y_{t}:$ The original time series value at time t,
-
$\theta_{1}$ : Theta coefficient for the original series,
-
$\theta_{2}$ : Theta coefficient for the trend component,
-
${\left\{\text{Trend}\right\}}_{t}$ : The extracted trend at time t,
- In the Theta approach, different values of *θ* (like θ = 0, θ = 2) are used to generate separate Theta lines, which are then extrapolated and combined.

#### 3.2.7 Seasonal and trend decomposition using LOESS (STL)

It is a robust and versatile method for decomposing time series data into its fundamental components: trend, seasonal, and remainder (residual) components. Introduced by Cleveland et al. (1990), STL employs locally weighted regression (Loess) to estimate both the trend and seasonal components, allowing it to adapt to changes over time. This decomposition helps in understanding underlying patterns and improving forecasting accuracy. Unlike classical decomposition methods, STL can effectively handle missing values, changing seasonality, and outliers.

The STL method has been widely adopted across various fields due to its adaptability and effectiveness. Hyndman and Athanasopoulos (2021) highlight STL’s capability to handle any type of seasonality, making it a preferred choice over classical decomposition methods. Furthermore, STL’s integration into statistical software packages, such as R’s forecast package, has facilitated its application in practical forecasting scenarios.

STL model formula:


$$Y_{t}=T_{t}+S_{t}+R_{t}$$ (8)

Where:

-
$Y_{t}$ is the observed value at time t.
-
$T_{t}$ represents the trend component.
-
$S_{t}\;$ denotes the seasonal component.
-
$R_{t}$ is the remainder (residual) component.

#### 3.2.8 Hybrid forecasting models

In addition to individual models, we employed a hybrid model, combining several of the above approaches to enhance predictive accuracy. Specifically, the methodology integrates auto. Arima, ets, thetam, nnetar, stlm, and tbats models, implemented using the forecast and smooth R packages. The auto.arima function automatically selects the best ARIMA model, while ets uses exponential smoothing for trend and seasonality. The thetam model combines exponential smoothing and autoregressive components, and nnetar utilizes a neural network for autoregressive forecasting. Stlm decomposes time-series data into seasonal, trend, and remainder components, and tbats handles complex seasonalities and trends. The hybrid model aggregates the outputs from these individual models, applying equal weights to each, providing a robust and accurate forecast by leveraging the strengths of each technique. This methodology offers a comprehensive approach to forecasting time-series data with varying patterns (Hyndman and Khandakar, 2008; Wang et al., 2013).

### 3.3 Model evaluation metrics

In this study, the accuracy of individual and hybrid models was evaluated on the in-sample performance (i.e., using the fitted one-step-ahead forecast). Forecast performance was evaluated with five widely used error metrics: Mean Error (ME), Mean Absolute Error (MAE), Root Mean Squared Error (RMSE), Mean Absolute Percentage Error (MAPE), and Mean Percentage Error (MPE). These measures are standard in forecasting research and are particularly useful for comparing different models on the same dataset (Hyndman and Koehler, 2006). Since each metric has its own strengths and limitations, we considered them collectively to ensure a more balanced evaluation of model performance (Perone, 2022). Model evaluation remains a critical but challenging step, as no single metric is universally appropriate across all contexts; the choice depends on data characteristics and the forecasting objectives.

The accuracy of individual and hybrid models was assessed using five commonly used error metrics:

i. Mean error (ME):

$$\mathrm{ME}=\frac{1}{n}\sum_{t=i}^{n}(y_{i}-\hat{y_{i}})$$ (9)

ii. Mean absolute error (MAE):

$$\mathrm{ME}=\frac{1}{n}\sum_{t=i}^{n}|y_{i}-\hat{y_{i}}|$$ (10)

iii. Root mean squared error (RMSE):

RMSE=1n∑t=in(−yi^)^2 (11)

iv. Mean absolute percentage error (MAPE) (Kim and Kim, 2016):

$$\text{MAPE}=\sum_{t=i}^{n}|\frac{\left\{y_{i}-\hat{y_{i}}\right\}}{y_{i}}|$$ (12)

v. Mean percentage error (MPE):

$$\;\mathrm{MPE}=\frac{100}{n}\sum_{t=i}^{n}\;\frac{\left\{y_{i}-\hat{y_{i}}\right\}}{y_{i}}$$ (13)

Where n is the number of observations $y_{i}$ represents the actual values and $\hat{y_{i}\;}$ are the predicted values.

## 4 Results and discussion

The descriptive analysis of the data shows that the average monthly Global Price of Cotton (2025) was $77.857 U. S. Cents per Pound with a standard deviation of $24.794. The lower and upper quartiles of the dataset were 60.44 and 86.98, respectively. The price range ranges from a minimum of $37.22 to a maximum of $229.67, with a median of $75.93. The data exhibits some degree of variability, with the minimum and maximum values indicating significant fluctuations over the observed period.

**Figure 1:**
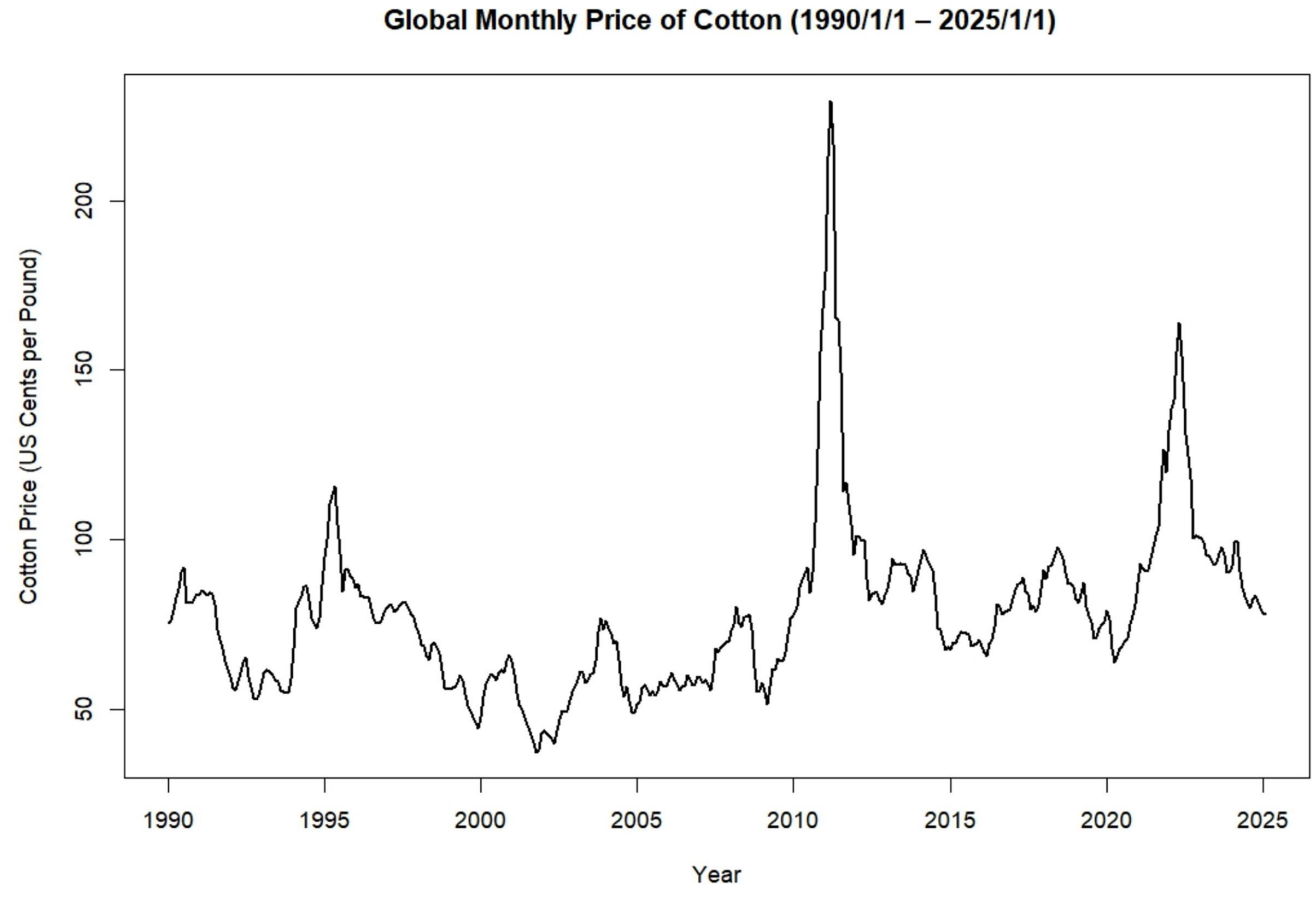
Time series plot of the monthly global price of cotton (1990/1/1–2025/1/1) (source: own work).

**Figure 2:**
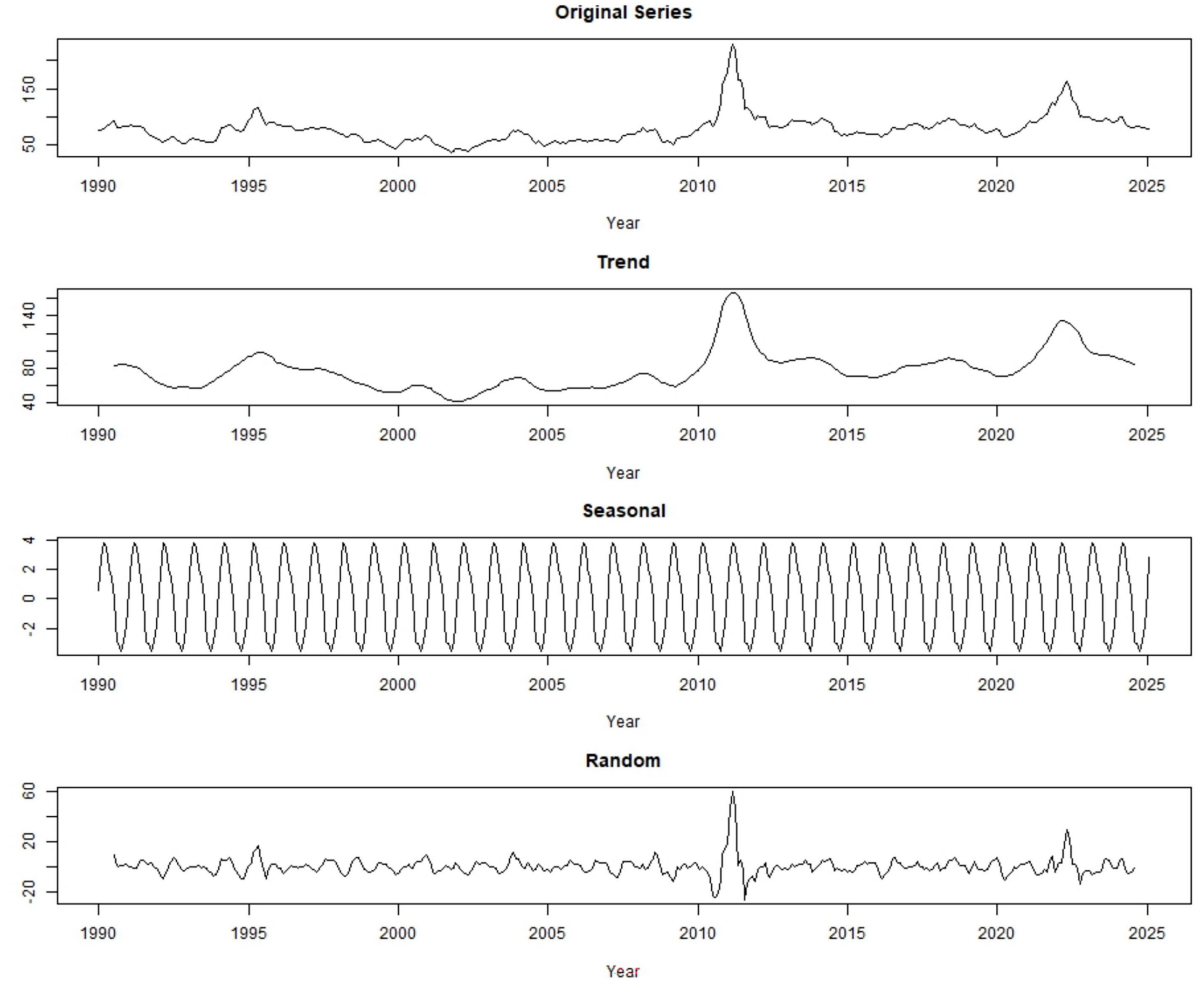
Additive time series decomposition showing observed data, trend, seasonal, and random components of global monthly cotton price (source: own work).

Table 1 and Figure 1 shows the trend in global monthly cotton prices from January 1990 to January 2025. Throughout this period, the time series exhibits clear fluctuations, with two major price peaks observed around 2010–11 and 2022–23. While certain intervals display relative stability, the overall pattern is characterized by variability and irregular movements in price (Figure 2).

**Table 1:** Summary statistics of the global monthly price of cotton (1990–2025).

Summary	Value
Minimum	$37.22
Maximum	$229.67
Mean	$77.857
Median	$75.93
Standard deviation	$24.794
Lower quartile	60.44
Upper quartile	86.98

Seasonal adjustment removes regular seasonal patterns from a time series to make the underlying trends and unexpected changes easier to see. By subtracting the seasonal effects from the original data, the adjusted series shows how values change over time without the usual ups and downs, highlighting long-term trends and unusual shifts.

**Figure 3:**
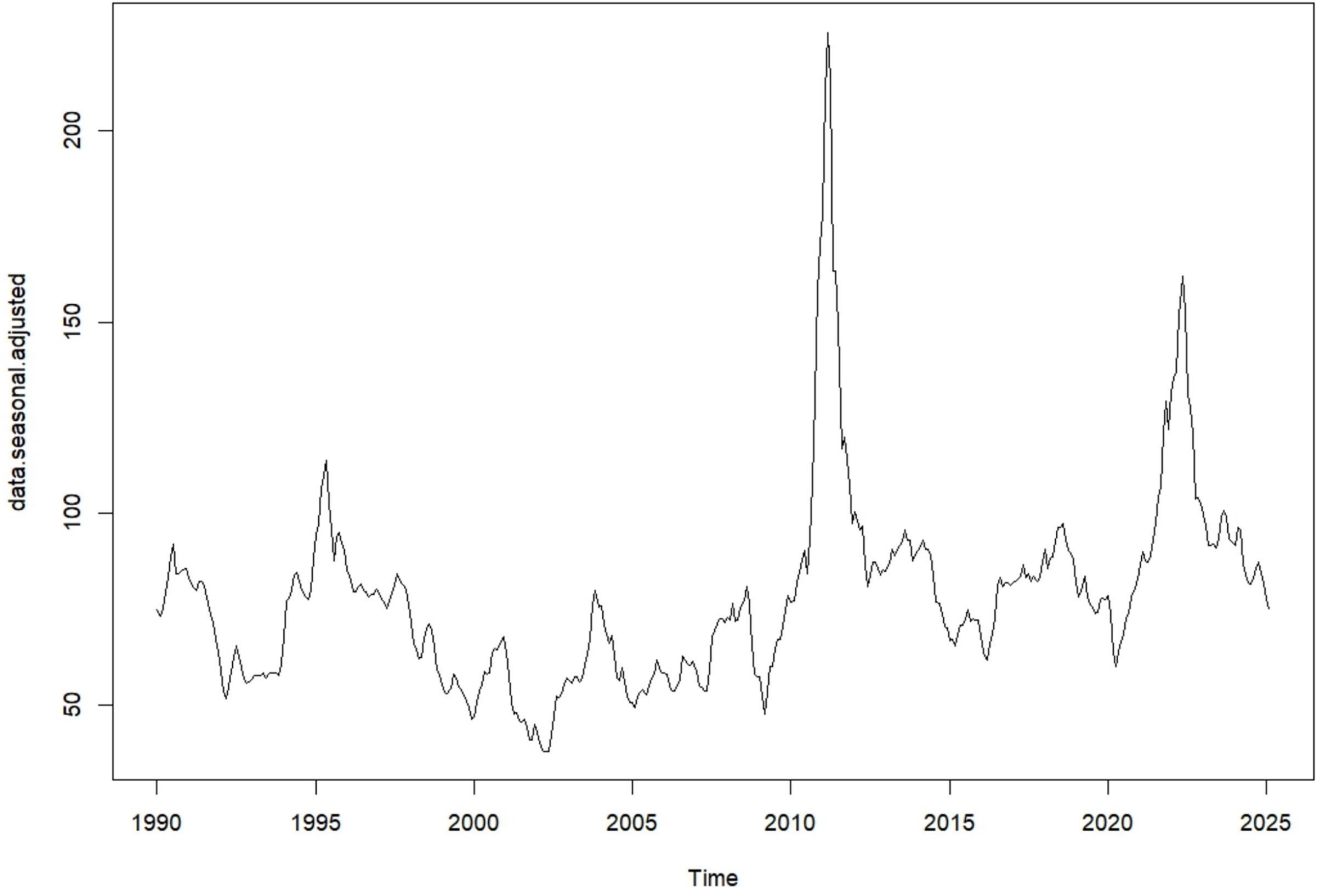
Seasonally adjusted time series plot of the global monthly cotton price (source: own work).

Figure 3 shows the data after removing recurring seasonal patterns, allowing the underlying trends and unusual fluctuations to be more clearly observed. By eliminating regular seasonal ups and downs, the adjusted series highlights long-term movements and unexpected changes, providing a clearer view of the true dynamics in the data over time.

The forecasting performance of various models was evaluated using multiple error metrics, including Mean Error (ME), Root Mean Square Error (RMSE), Mean Absolute Error (MAE), Mean Percentage Error (MPE), and Mean Absolute Percentage Error (MAPE). The results are presented in Table 2.

**Table 2:** Performance of individual and hybrid models based on multiple evaluation metrics for identifying the best-fit model.

Models	ME	RMSE	MAE	MPE	MAPE
EQUAL	0.042	4.264	2.576	−0.008	3.123
CV. ERRORS	0.043	4.290	2.591	−0.012	3.139
AUTO. ARIMA(4,1,1) (0,0,2) [12]	0.116	4.819	2.989	−0.086	3.626
ETS (M, Ad, N)	−0.014	5.182	3.044	0.017	3.711
NNAR (26,1,14) [12]	−0.008	***1.164***	***0.832***	−0.064	***1.192***
THETA	0.000	5.668	3.329	*−0.147*	4.014
STL +ETS (M, Ad, N)	−0.015	4.768	2.942	0.012	3.617
TBATS (0, 0, 0, −, −)	*−0.054*	5.007	3.047	−0.123	3.658
Hybrid 1 (ARIMA X ETS)	0.055	4.909	2.951	−0.038	3.589
Hybrid 2 (ARIMA X NNAR)	0.084	2.820	1.875	−0.041	2.336
Hybrid 3 (ARIMA X TBATS)	0.029	4.858	2.971	−0.105	3.582
Hybrid 4 (ARIMA X STL)	0.055	4.650	2.828	−0.040	3.443
Hybrid 5 (ETS X NNAR)	0.013	2.968	1.883	0.012	2.351
Hybrid 6 (ETS X TBATS)	−0.036	5.004	2.996	−0.059	3.625
Hybrid 7 (ETS X STL)	−0.014	4.916	2.946	0.014	3.596
Hybrid 8 (NNAR X TBATS)	−0.006	2.947	1.923	−0.059	2.378
Hybrid 9 (NNAR X STL)	0.013	2.875	1.893	0.015	2.377
Hybrid 10 (TBATS X STL)	−0.036	4.771	2.887	−0.061	3.493
Hybrid 11 (ARIMA X ETS X NNAR)	0.064	3.465	2.168	−0.002	2.665
Hybrid 12 (ARIMA X ETS X STL)	0.060	3.997	2.450	0.022	3.000
Hybrid 13 (ARIMA X ETS X STL)	0.033	4.791	2.869	−0.022	3.494
Hybrid 14 (ARIMA X ETS X NNAR X STL X TBATS)	0.042	4.110	2.509	0.003	3.051

The bold values of RMSE (1.164), MAPE (1.192%), and MAE (0.832) for the NNAR (26,1,14) [12] model showed minimal error and the highest accuracy among all the models, indicating the best overall performance. The italics values showed minimal error and the highest accuracy among all the models, indicating the best overall performance.

### 4.1 Model performance

Table 2 summarizes the forecasting performance of both individual and hybrid models based on the evaluation using ME, RMSE, MAE, MPE, and MAPE. Among all models, the NNAR (26,1,14) [12] model showed the best overall performance, achieving the lowest RMSE (1.164), MAPE (1.192%), and MAE (0.832). This indicates its strong predictive accuracy and ability to effectively capture nonlinear and seasonal patterns in global cotton prices. The model used 26 lagged observations and 14 neurons in a single hidden layer, with a seasonal period of 12 to represent annual variations.

In contrast, the THETA model performed the weakest, recording the highest RMSE (5.668), MAE (3.329), and MAPE (4.014%), reflecting poor predictive capability. Among the hybrid models, Hybrid 6 (ETS × TBATS) had the largest errors (RMSE = 5.004, MAE = 2.996, MAPE = 3.625%), while Hybrid 2 (ARIMA × NNAR) showed the highest mean error (ME = 0.084), suggesting a stronger systematic bias. Although combining multiple modeling techniques, the hybrid models did not enhance forecast accuracy and generally underperformed relative to the NNAR model.

The results indicate that the global cotton price series was primarily driven by nonlinear seasonal dynamics, which were effectively captured by the NNAR model. This suggests that neural network-based approaches are well-suited for modeling commodity price behavior when nonlinearities dominate. However, hybrid models may offer additional advantages in more complex or highly volatile datasets, particularly when both linear and nonlinear components jointly influence price movements. By combining complementary modeling techniques, hybrid approaches can improve resilience and accuracy in the presence of noise, sudden structural changes, or shifts in market regimes.

**Figure 4:**
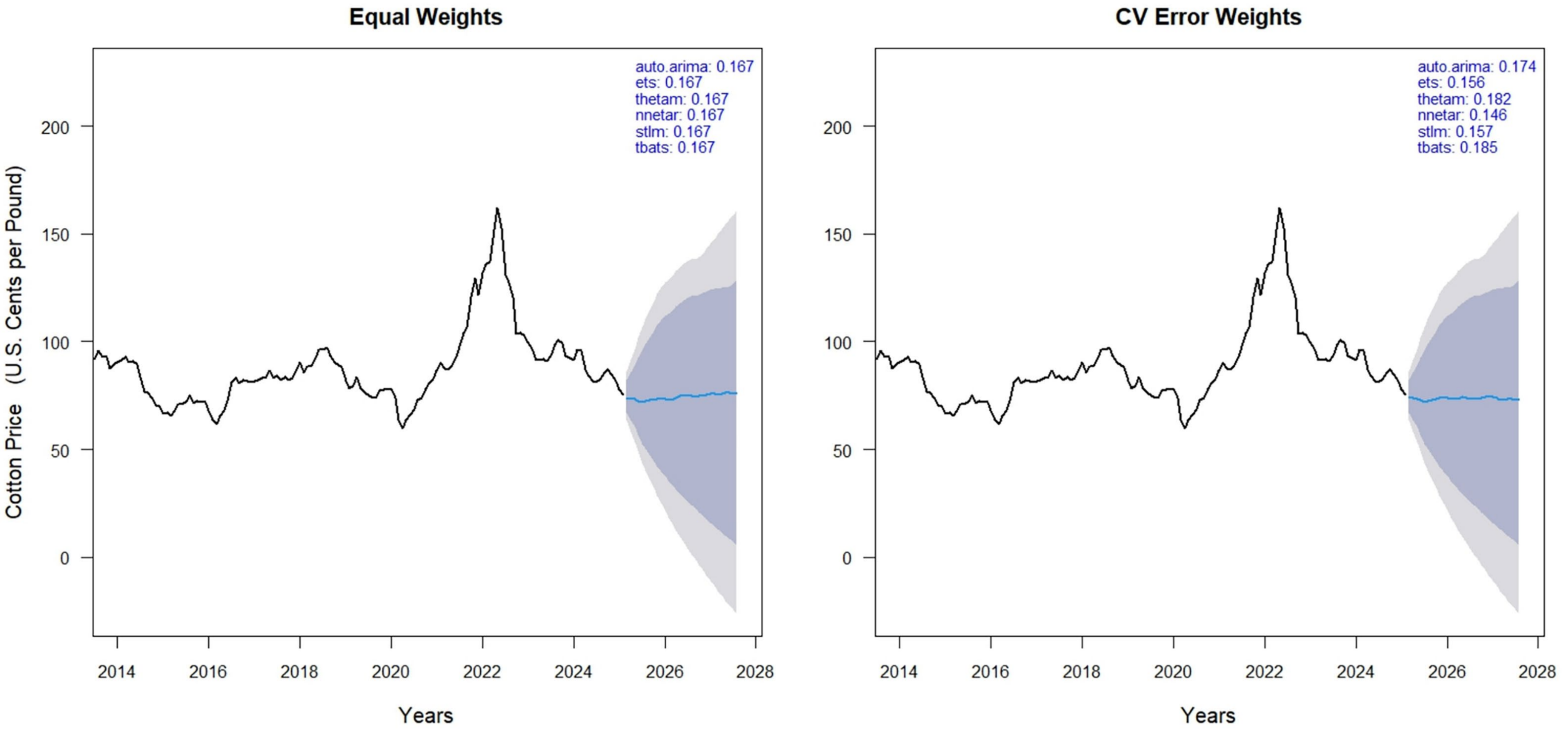
Forecasts of global monthly cotton prices using model averaging approaches.

**Figure 5:**
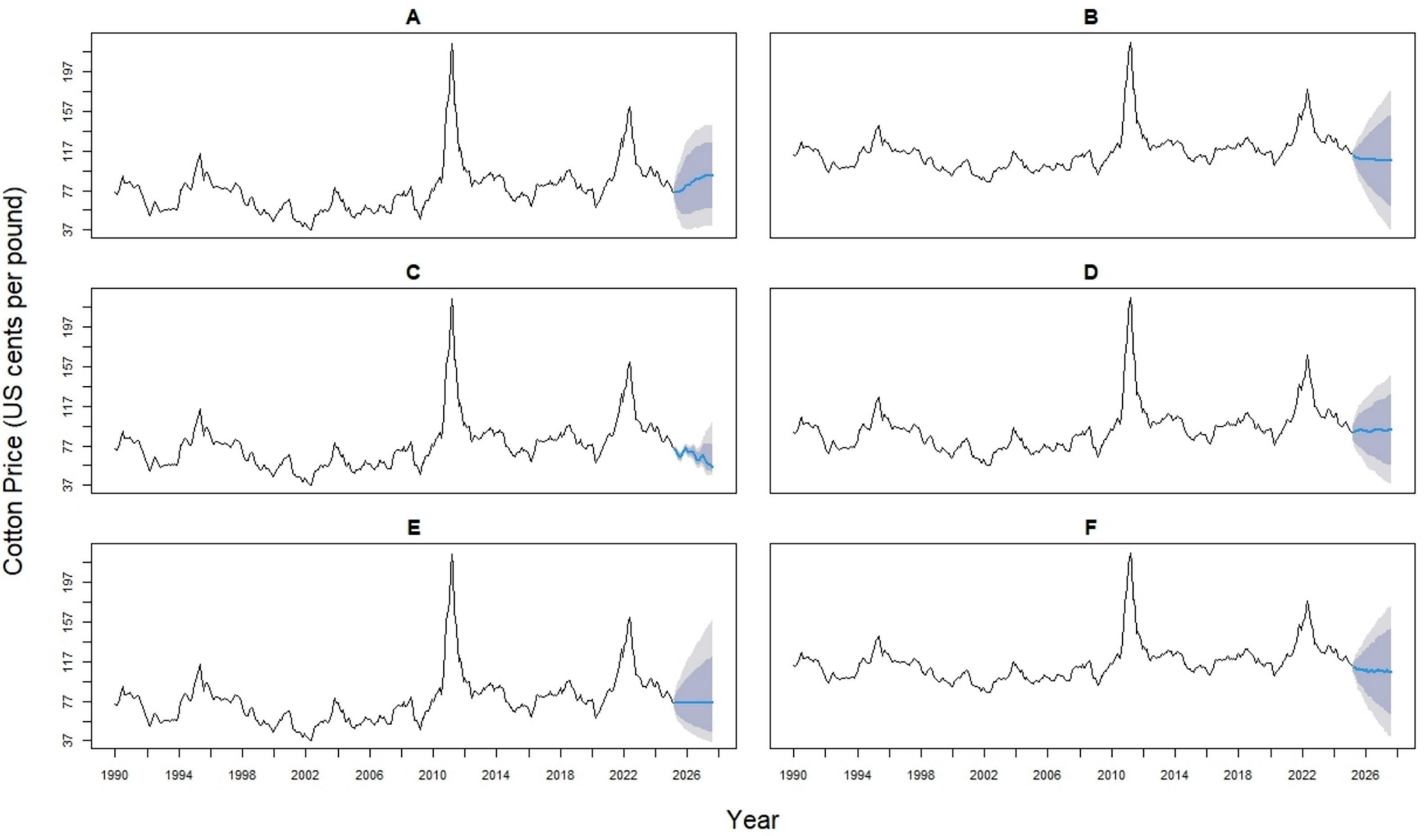
Time series forecasts of global monthly cotton prices (in U. S. cents per pound) using six forecasting models **(A–F)**. **(A)** ARIMA (4,1,1) (0,0,2) [12] model; **(B)** ETS (M, Ad, N) model; **(C)** NNAR (26,1,14) [12] model; **(D)** Theta model; **(E)** TBATS (0,0,0, −, −) model; **(F)** STL + ETS (M, Ad, N) model. The black lines represent historical observations, blue lines show model forecasts, and shaded areas indicate prediction intervals.

In Figure 4 left panel displays forecasts obtained by equal weighting the six models (ARIMA, ETS, ThETAM, NNETAR, STLM, TBATS), while the right panel shows forecasts using cross-validation (CV) error-based weights. Weights of individual models are shown in the top right corner of each panel, reflecting their contribution to the hybrid forecast. Equal weighting results in uniform contributions (0.167 each), whereas CV error weighting adjusts contributions based on model accuracy, emphasizing models with lower prediction error. The black line represents historically observed cotton prices, and the blue line indicates the model forecast. Compared to equal weighting, the CV-based method provides a data-driven weighting structure that may improve forecast reliability depending on model performance (Figure 5).

- The AUTO. ARIMA(4,1,1) (0,0,2) [12] model, with a weight of 0.171 in CV-based averaging, showed moderate price fluctuations and increasing forecast uncertainty over time. It’s higher error values (RMSE = 4.819, MAE = 2.989, MAPE = 3.626) compared to the NNAR model indicate less accurate predictions and struggles with nonlinear and complex patterns in cotton prices.
- The ETS (M, Ad, N) model, assigned a weight of 0.154 in CV-based model averaging, underperformed relative to the NNAR model, as indicated by higher error metrics (RMSE = 5.182, MAE = 3.044, MAPE = 3.711). This underperformance is likely due to the model’s lack of a seasonal component and it’s limited ability to capture nonlinear and complex patterns present in cotton prices.
- Among all the models, the NNAR (26,1,14) [12] model, which received a weight of 0.159 in cross-validated model averaging, emerged as the best performer. It achieved the lowest evaluation metrics: RMSE (1.164), and MAE (0.832), indicating the smallest average error magnitudes, and MAPE (1.192) showing minimal relative percentage errors, with minimal bias (ME = −0.008, MPE = −0.064). By effectively capturing nonlinear patterns and short-term seasonal fluctuations in global monthly cotton prices, the NNAR model provided stable forecasts with a slight downward adjustment.
- The THETA model, a simple decomposition-based method, was assigned a weight of 0.179 in cross-validated model averaging. Its bias metrics (ME = 0.000, MPE = −0.147) indicate minimal systematic error; however, its overall forecast accuracy is limited, as reflected by the highest RMSE (5.668), MAE (3.329), and MAPE (4.014) among all models. These results highlight that simplistic decomposition methods are inadequate for modeling complex, nonlinear time series as compared to the NNAR model.
- The STLM [STL + ETS (M, Ad, N)] model, assigned a weight of 0.155 in cross-validated model averaging, exhibited a minor under prediction bias (ME = −0.015, MPE = −0.123). However, its forecast accuracy was lower than that of the NNAR model, as reflected by higher error values (RMSE = 4.768, MAE = 2.942, MAPE = 3.617). This performance suggests that the STLM model is less capable of capturing complex nonlinear dynamics and short-term fluctuations in the global cotton price series, likely due to its sensitivity to historical seasonal spikes, which contributed to increased short-term forecast volatility.
- The TBATS (0,0,0, −, −) model, with a cross-validated weight of 0.182, exhibited minimal bias (ME = −0.054, MPE = −0.123). However, its relatively higher errors compared to the NNAR model (RMSE = 5.007, MAE = 3.047, MAPE = 3.658) indicate difficulty in capturing the fluctuations of cotton prices. Its lack of seasonal or complex components likely limits its overall accuracy.

### 4.2 Hybrid models

**Figure 6:**
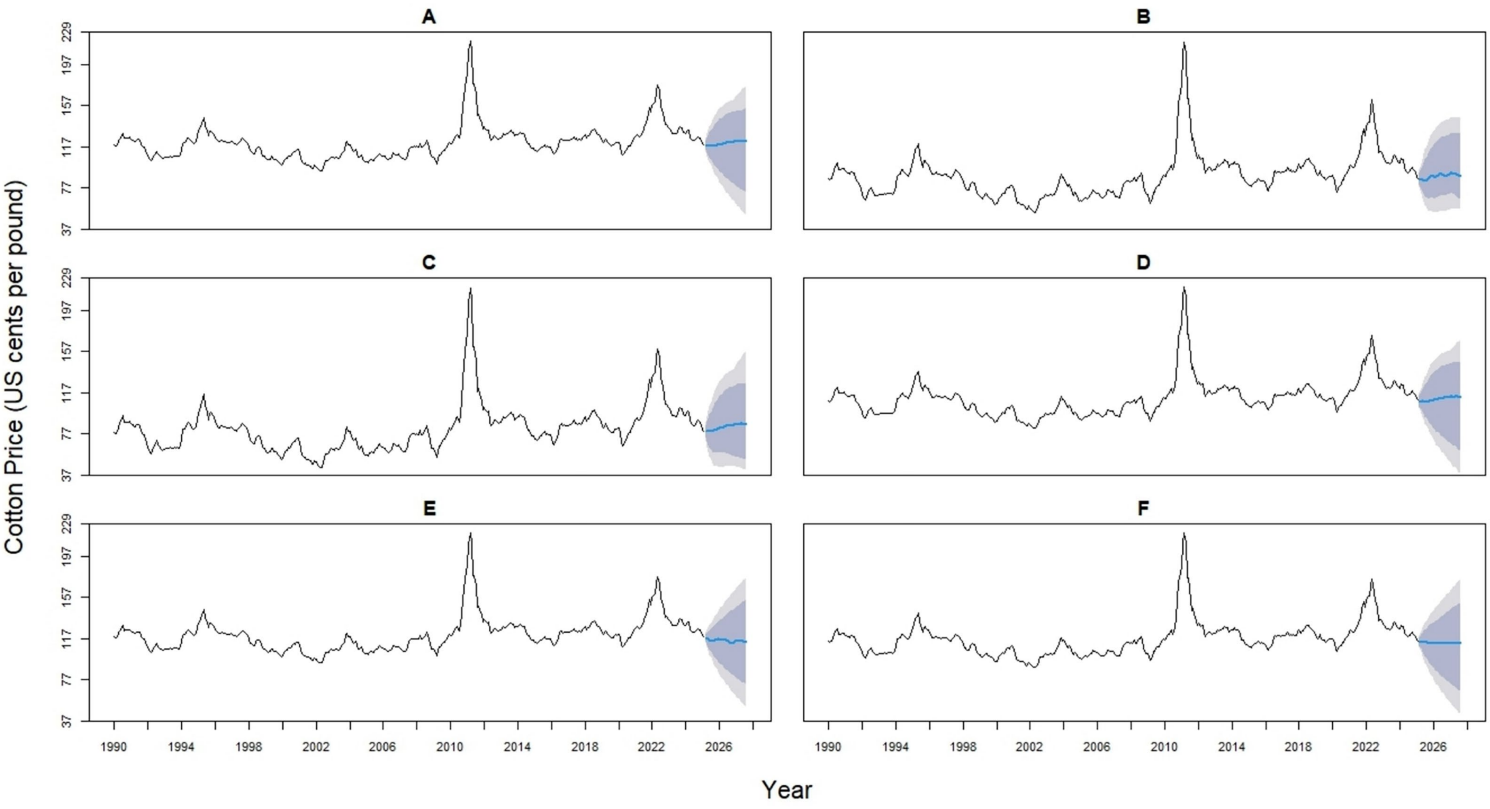
Forecast comparison of six hybrid models for monthly global cotton prices. Each panel displays one hybrid model (Hybrid 1–6, labeled **A–F**), formed by combining two base models (ARIMA, ETS, NNAR, Theta, TBATS, or STL) with varying weights. Historical prices are shown in black, and forecasts are in blue. Shaded regions represent 80 and 95% prediction intervals, illustrating the uncertainty in the forecasts.

**Figure 7:**
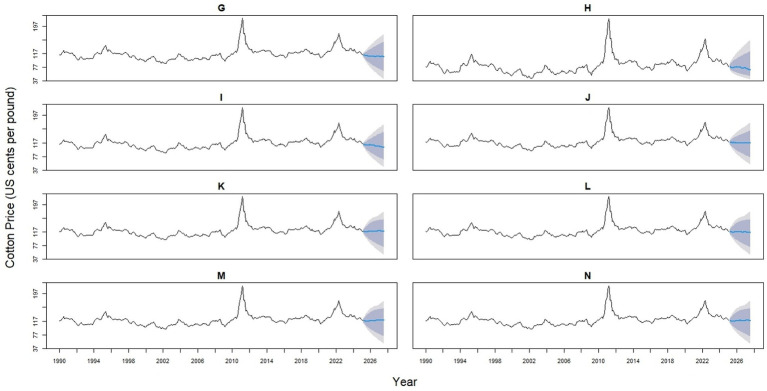
Forecast comparison of eight hybrid models for global monthly cotton prices. Each panel displays one hybrid model (Hybrid 7–14, labeled **G-N**), formed by combining two base models (ARIMA, ETS, NNAR, Theta, TBATS, or STL) with varying weights. Historical prices are shown in black, and forecasts are in blue. Shaded regions represent 80 and 95% prediction intervals, illustrating the uncertainty in the forecasts.

The 14 hybrid models represent a spectrum of statistical and machine learning integrations, designed to enhance predictive accuracy by capturing both linear and nonlinear patterns. Hybrids 1–4 combine ARIMA with ETS (0.53/0.47), NNAR (0.514/0.486), TBATS (0.485/0.515), or STL (0.525/0.475). Hybrids 5–7 pair ETS with NNAR (0.491/0.509), TBATS (0.458/0.542), or STL (0.499/0.501), while Hybrids 8–9 integrate NNAR with TBATS (0.431/0.569) or STL (0.499/0.501). Hybrid 10 combines TBATS (0.499) with the STL (0.501) model. Likewise, Hybrid 11 blends ARIMA (0.364), ETS (0.327), and NNAR (0.312). Hybrid 12 combines ARIMA (0.267), ETS (0.240), and STL (0.241). Hybrid 13 incorporates ARIMA (0.357), ETS (0.321), and STL (0.322), whereas Hybrid 14 provides the most complex structure, balancing ARIMA (0.212), ETS (0.191), NNAR (0.180), STL (0.192), and TBATS (0.225) (Figures 6, 7).

Among the hybrid models, those incorporating NNAR, particularly Hybrid 2 (ARIMA × NNAR), showed lower error values (RMSE = 2.820, MAE = 1.875, MAPE = 2.336) compared with other hybrids. However, these errors remained higher than those of the standalone NNAR model (RMSE = 1.164, MAE = 0.832, MAPE = 1.192). Therefore, the NNAR model proved to be the best-fitting model, demonstrating the lowest error metrics and the highest predictive accuracy.

In our study, the standalone NNAR model achieved lower error values than the hybrid models, highlighting its strong ability to capture nonlinear patterns in cotton price dynamics. This aligns with the findings of Taskaya-Temizel and Casey (2005), who reported that combining multiple models for forecasting does not always lead to better accuracy and can sometimes perform worse than individual models.

However, the superior performance of NNAR in our dataset does not diminish the value of hybrids. Forecast combinations are widely recognized as a means of improving robustness and stability across different time horizons and market conditions, even when a single model performs best in a specific setting (Makridakis et al., 2018). Hybridization remains valuable for enhancing resilience under uncertainty and mitigating the risk of model-specific weaknesses. As noted by Zhang (2003), hybrid models tend to outperform single models when the series exhibits a mix of linear and nonlinear patterns, because each component is specialized in capturing distinct aspects of the data. In our case, however, the cotton price series appears to be dominated by nonlinear patterns, which the NNAR model was able to capture more effectively than the hybrid configuration.

In this study, 22 forecasting models were evaluated to assess their effectiveness in predicting monthly cotton prices. The models included both statistical and machine learning approaches, namely Autoregressive Integrated Moving Average (ARIMA), Exponential Smoothing State Space (ETS), Neural Network Autoregression (NNAR), Theta Model, TBATS, and Seasonal-Trend Decomposition using LOESS (STL). Given the complex and nonlinear dynamics of cotton prices, identifying the most accurate and reliable forecasting approaches is critical for informed decision-making by stakeholders. By systematically comparing these models, this study addresses the need to evaluate both traditional and machine learning-based methods within a unified framework, providing insights into which techniques best capture the temporal patterns of cotton markets.

### 4.3 Model interpretability: best fit model

A Neural Network Autoregressive (NNAR) model with the configuration NNAR (26, 1, 14)12 was employed. This model utilized 26 lagged observations as inputs and 14 neurons in a single hidden layer to model nonlinear dependencies within the data. The seasonal period of 12 was again used to capture annual patterns. The NNAR model demonstrated superior predictive performance compared to all other models considered. It achieved the lowest values for Root Mean Square Error (RMSE) and Mean Absolute Error (MAE), indicating a high level of accuracy and minimal prediction error. Additionally, the model recorded the lowest Mean Absolute Percentage Error (MAPE) of 1.20325, underscoring its effectiveness in reducing relative forecast errors.

**Figure 8:**
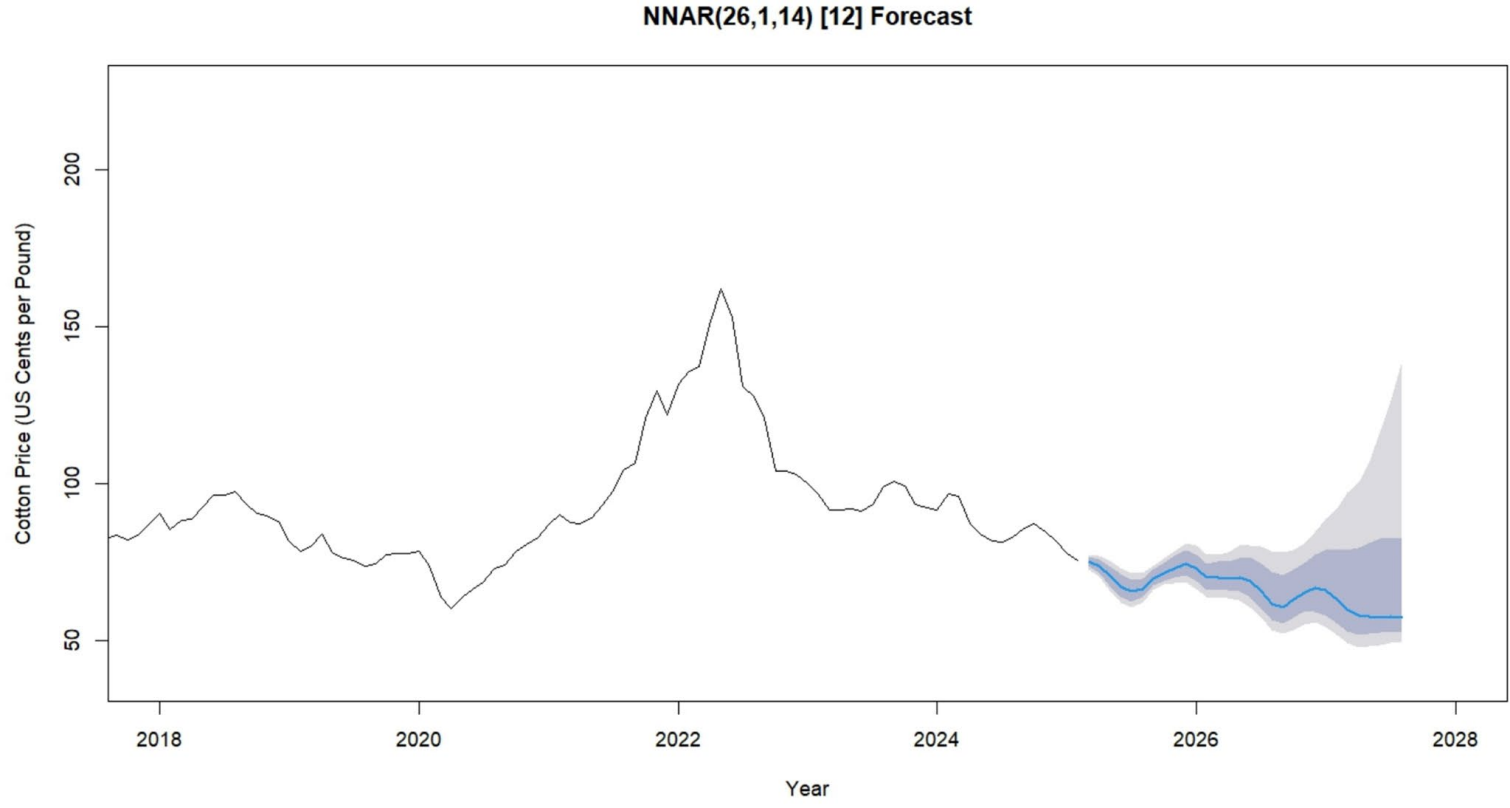
A 30-month future forecasted monthly global cotton prices using NNAR (26,1,14) [12]: best fit model.

Figure 8 presents a 30-month forecast of cotton prices generated by the NNAR (26,1,14) [12] model. The x-axis shows time in months, while the y-axis represents predicted cotton prices in U. S. cents per pound. The model uses the previous 26 months of data as input lags, applies one seasonal difference, and includes 14 hidden neurons to capture nonlinear patterns. The forecast indicates moderate fluctuations, with prices expected to vary between 66 and 74 cents per pound. These cyclical movements reflect potential periods of price increases (peaks) and declines (troughs), capturing both seasonal and market-driven dynamics. As highlighted by Gneiting et al. (2007), effective probabilistic forecasting requires a balance between sharpness (narrowness of intervals) and calibration (ensuring that the actual values fall within the predicted range). In this context, the NNAR model provides not only point forecasts but also a clear measure of uncertainty, enabling stakeholders in the cotton sector to make informed, resilient decisions in the face of market variability.

In our study, the NNAR model’s superior performance in forecasting cotton prices, as supported by its lowest RMSE, MAE, and MAPE, highlights the effectiveness of machine learning techniques in capturing the complex and nonlinear dynamics of agricultural commodity markets. This is attributed to the model’s ability to uncover hidden patterns and nonlinear dependencies in the data.

Based on this, Khashei and Bijari (2010) further emphasized the utility of artificial neural networks (ANNs) in time series forecasting, highlighting their adaptability and ability to model complex relationships. This flexibility makes ANNs particularly effective for real-world forecasting problems, which often involve uncertainty and nonlinearity. Among these models, Neural Network Autoregressive (NNAR) models have gained recognition for their reliability, with Zhang (2003) emphasizing their strength in capturing nonlinear trends and seasonal patterns often observed in economic and financial time series. Several studies have demonstrated the applicability and effectiveness of the NNAR model in forecasting a wide range of commodities, validating their performance through various error metrics that assess forecasting accuracy. In the study done by Reza and Debnath (2025), NNAR (5,1,10) [12] and NNAR (3,1,10) [12] models were applied to forecast the prices of wheat and rice, two key agricultural commodities. The models effectively captured both short-term patterns and complex relationships, with performance measured using standard accuracy metrics like RMSE, MAE, MASE, and MAPE. This study further supports the practical value of NNAR models in agricultural forecasting contexts. Similarly, Raghav et al. (2022) used the NNAR (1,1) model to forecast pulse production. Although simpler in design, the model was assessed using a broad set of evaluation metrics: Mean Error (ME), RMSE, MAE, Mean Percentage Error (MPE), MAPE, and MASE. This comprehensive evaluation helped ensure the model’s reliability in capturing the variability and irregularities typical of agricultural production data. Similar results were reported by Almarashi et al. (2024a), where the NNAR model outperformed other competing models, including ARIMA, ETS, and TBATS, based on lower values of RMSE, MAE, and MAPE. These findings suggest that the NNAR model is a more effective approach for time series forecasting compared to conventional methods. Likewise, Melina et al. (2024) applied the NNAR (1,10) model to forecast gold prices. With a single lag input and a higher number of hidden neurons, this model was well-equipped to capture complex nonlinear dependencies in volatile financial data. Performance was assessed using RMSE and MAE, metrics especially relevant in financial forecasting, where even small errors can have substantial consequences.

The robustness of the Neural Network Auto-Regressive (NNAR) model extends beyond agricultural and financial applications. For instance, Qureshi et al. (2025) showed that NNAR effectively predicted cardiovascular disease mortality, while Alshanbari et al. (2023) found it successful in forecasting COVID-19 cases and recoveries in Pakistan. These studies underscore NNAR’s ability to capture complex nonlinear patterns in diverse time series datasets, suggesting its potential as a robust tool for forecasting agricultural prices, such as cotton, which frequently exhibit similar nonlinear dynamics. Furthermore, hybrid approaches that combine NNAR with nonlinear models and residual volatility modeling, as proposed by Iftikhar et al. (2025) for stock indices, could help improve forecast accuracy and reliability in commodity markets. Despite these strengths, NNAR and hybrid methods remain less transparent than traditional statistical models, emphasizing the need for further methodological refinement to improve interpretability and practical applicability in agricultural economics. Overall, these studies collectively validate the NNAR model’s adaptability across different forecasting contexts. This shows that NNAR is a reliable and flexible tool for finding both simple and complex patterns in different forecasting situations.

### 4.4 Practical implications

Historical events illustrate the sensitivity of cotton markets to global dynamics. In Figure 1, the sharpest rise in global cotton prices happened in early 2011, reaching over $2.00 per pound, the highest in more than 100 years. This spike came after cotton demand began to recover following the 2008–2009 economic crisis. The surge was caused by a mix of factors, including a shortage of cotton and heavy trading (Agarwal and Narala, 2020). The supply shortage was worsened by poor harvests in key producing countries, extreme weather events, export restrictions, and rising global demand (Janzen et al., 2018). Weather events like floods, droughts, and heavy rains caused major disruptions in cotton production, with yields varying by 10–80% (Hoerling et al., 2013). In the U. S., the worst drought in over a century between mid-2010 and September 2011 greatly impacted cotton production (Anyamba et al., 2014). After prices rose above 200 cents per pound post-2011, the demand for cotton dropped, and people began using more Man-Made Fibers (MMF). However, when prices stabilized between 90 and 100 cents per pound from 2012 to 2014, cotton became more competitive again against MMF and other non-cotton textiles (Agarwal and Narala, 2020). This period highlights the importance of monitoring the cotton market and taking timely actions to reduce future price swings.

Most recently, another significant fluctuation in global cotton prices observed between 2020 and 2022 can largely be attributed to two major global disruptions: the COVID-19 pandemic and the Russia-Ukraine conflict. The pandemic severely disrupted global supply chains, particularly in sectors like textiles, leading to both reduced demand and increased production costs. Early in the pandemic, lockdowns and economic uncertainty caused a sharp drop in textile demand, resulting in lower cotton prices (Emran and Schmitz, 2023). At the same time, supply chain disruptions made it harder to get important materials and caused delivery delays, which increased the cost of producing textiles (Cai and Luo, 2020). The COVID-19 pandemic resulted in shortages and disruptions in the production and transportation of raw materials (Paul et al., 2021). Despite this initial downturn, the cotton market began to recover by the third quarter of 2020 as global demand gradually resumed. Prices continued to rise through 2021, eventually peaking in June 2022. These prices movements reflect how closely cotton markets are tied to broader global dynamics and supply chain conditions (Erkencioglu et al., 2023). In addition, the Russia-Ukraine conflict introduced further instability by disrupting global trade flows and raising input and import costs. These pressures indirectly increased the cost of cotton production and added uncertainty to global cotton markets (Arndt et al., 2023). Together, these events underscore the vulnerability of agricultural commodity markets to global crises and the importance of adaptable forecasting models in such volatile contexts.

## 5 Conclusion

Agricultural commodity prices are shaped by complex global and local supply–demand dynamics. Timely, accurate forecasts help stakeholders make informed decisions and manage price-related risks. This study examined the predictive performance of various univariate time series forecasting models for global monthly cotton prices using data from January 1990 to January 2025. The NNAR (26,1,14) [12] model demonstrated superior accuracy in forecasting global monthly cotton prices, in comparison to several individual models (ARIMA, ETS, STL, TBATS, Theta, and NNAR), as well as hybrid models combining these methods, validated by the key evaluation metrics like RMSE, MAE, and MAPE. The NNAR model’s ability to capture both linear and nonlinear trends, as well as seasonal variations, highlights its suitability for modeling complex time series data. Given that each forecasting method has distinct strengths and limitations, model selection should be guided by the nature of the data and the specific forecasting goals. In some cases, combining models may help further reduce prediction errors. Our analysis also highlights the sensitivity of global cotton prices to macroeconomic shocks, supply chain disruptions, and external events. For instance, the sharp price spike in 2011 and the volatility seen between 2020 and 2022 reflect the broader impact of such factors. These findings emphasize the need for reliable forecasting tools to support informed decision-making, risk management, and strategic planning in the agricultural sector. This study was limited to univariate time series models, without considering multivariate approaches. Future research could incorporate relevant covariates, such as weather, trade policies, and economic indicators, to improve forecasting accuracy. The use of advanced machine learning and hybrid methods, including deep learning, Random Forest, and Support Vector Regression, holds strong potential for improving predictive performance. Developing such models and aligning them with real-world applications can help the agricultural sector better anticipate market changes, enhancing price stability and resilience. Furthermore, adopting a rolling-origin (out-of-sample) evaluation approach provides additional validation of forecasting accuracy.

## Data Availability

Publicly available datasets were analyzed in this study. This data can be found at: https://fred.stlouisfed.org/series/PCOTTINDUSDM.

## Footnotes

1
https://fred.stlouisfed.org/series/PCOTTINDUSDM
